# Supplementary material for: Unraveling the Genetic Control of Pigment Accumulation in Physalis Fruits
Source: Int J Mol Sci. 2024 Sep 12;25(18):9852. doi: 10.3390/ijms25189852 (PMC11432741; doi:10.3390/ijms25189852)
Supplement: Supplementary file 1 [file ijms-25-09852-s001.zip › ijms-3173525-supplementary.pdf]

Supplementary Table S1 Quality control of sequencing data of *P. pubescens(a) and p. alkekengi (b).*

Sample	Raw Data		Valid Data		Valid	Q20%	Q30%	GC
	Read	Base	Read	Base	Ratio(reads)	Q20%	Q30%	content
Y_S1_1	39545396	5.93G	38317696	5.75G	96.90	99.94	97.97	45
Y_S1_2	36472646	5.47G	35255978	5.29G	96.66	99.95	98.00	44.50
Y_S1_3	39530152	5.93G	38219868	5.73G	96.69	99.94	97.95	44
Y_S2_1	40199206	6.03G	38739204	5.81G	96.37	99.97	98.03	42.50
Y_S2_2	40846614	6.13G	38688670	5.80G	94.72	99.97	98.10	42
Y_S3_3	41918926	6.29G	40402050	6.06G	96.38	99.96	97.86	42.50
Y_S3_1	39083568	5.86G	37740510	5.66G	96.56	99.98	98.02	43
Y_S3_2	40076132	6.01G	38653066	5.80G	96.45	99.97	98.05	42.50
Y_S3_3	41918926	6.29G	40402050	6.06G	96.38	99.96	97.86	42.50
Y_S4_1	39938908	5.99G	38477192	5.77G	96.34	99.96	97.85	42.50
Y_S4_2	41874236	6.28G	40350130	6.05G	96.36	99.96	97.89	42.50
Y_S4_3	42182070	6.33G	40745290	6.11G	96.59	99.96	97.76	42

(a)

Sample	Raw Data		Valid Data		Valid	Q20%	Q30%	GC
	Read	Base	Read	Base	Ratio(reads)	Q20%	Q30%	content
R_S1_1	41612410	6.24G	40538008	5.66G	97.42	97.61	92.77	42.91
R_S1_2	43216840	6.48G	42128502	5.89G	97.48	97.65	92.85	42.70
R_S1_3	43023762	6.45G	41854332	5.85G	97.28	97.59	92.72	42.84
R_S2_1	41160578	6.17G	40086186	5.60G	97.39	97.57	92.67	43.26
R_S2_2	43293706	6.49G	42316804	5.92G	97.74	97.68	92.90	43.20
R_S2_3	42669018	6.40G	41587068	5.82G	97.46	97.92	93.58	42.98
R_S3_1	43602764	6.54G	42391172	5.92G	97.22	97.79	93.30	42.54
R_S3_2	43036602	6.46G	41850482	5.85G	97.24	97.77	93.25	42.56
R_S3_3	41696862	6.25G	40567132	5.67G	97.29	97.82	93.35	42.50
R_S4_1	42662452	6.40G	41525760	5.81G	97.34	97.91	93.58	42.34
R_S4_2	44473986	6.67G	43194788	6.04G	97.12	97.81	93.36	42.26
R_S4_3	43202744	6.48G	42010544	5.87G	97.24	97.81	93.33	42.29

(b)

Instruction: Supplementary Table S1 presents the sequencing data results for *P. pubescens* (a) and *P. alkekengi*.

Supplementary Table S2 Summary table of gene function annotation of *p. alkekengi*.

DB	Num	Ratio(%)
All	59393	100.00
GO	24886	41.90
KEGG	20217	34.04
Pfam	23917	40.27
swissprot	20534	34.57
eggNOG	30824	51.90
NR	30968	52.14

Instruction: Supplementary Table S2 shows a comparison of the Unigenes sequences from *P. alkekengi* with gene sequences from various databases, including NCBI_nr, GO, KEGG, Pfam, Swiss-Prot, and EggNog. The results indicate that a total of 59,393 genes were annotated in *P. alkekengi.*
